# The Effect of Sex on the Chemical and Mineral Composition of the Meat, Bone and Liver of Giraffe (*Giraffa giraffa angolensis*)

**DOI:** 10.3390/foods13030394

**Published:** 2024-01-25

**Authors:** Louwrens Christiaan Hoffman, Bianca L. Silberbauer, Tersia Needham, Daniel Bureš, Radim Kotrba, Philip E. Strydom

**Affiliations:** 1 Department of Animal Sciences, University of Stellenbosch, Private Bag X1, Matieland, Stellenbosch 7602, South Africa; biancasilberbauer95@gmail.com (B.L.S.); pestrydom@sun.ac.za (P.E.S.); 2 Centre for Nutrition and Food Sciences, Queensland Alliance for Agriculture and Food Innovation (QAAFI), The University of Queensland, Digital Agricultural Building, Gatton 4343, Australia; 3 Department of Animal Science and Food Processing, Faculty of Tropical AgriSciences, Czech University of Life Sciences Prague, Kamýcká 129, Suchdol, 16500 Prague, Czech Republic; needham@ftz.czu.cz (T.N.); kotrba@ftz.czu.cz (R.K.); 4 Department of Food Science, Faculty of Agrobiology Food and Natural Sciences, Czech University of Life Sciences Prague, Kamýcká 129, Suchdol, 16500 Prague, Czech Republic; bures.daniel@vuzv.cz; 5 Department of Ethology, Institute of Animal Science, Přátelství 815, Praha Uhříněves, 10400 Prague, Czech Republic

**Keywords:** game meat, giraffe, muscle, liver, bone, lipid content, moisture, ash, protein, mineral

## Abstract

Consumers tend to buy meat based on visual physical characteristics, which are affected by the chemical composition of the meat, and there is very little known about the chemical composition of the meat of giraffe. This study therefore aims to broaden the knowledge base on the chemical composition of giraffe meat, rib bone and liver. Eight different muscles from 15 giraffes were analyzed to determine the chemical composition, yielding an average moisture of 77.2 ± 0.09 g/100 g meat, an average protein of 20.8 ± 0.09 g/100 g meat, an average intramuscular fat (IMF) of 1.4 ± 0.03 g/100 g meat and an average ash of 1.1 ± 0.01 g/100 g meat. There was a significant interaction between sex and muscle for the moisture, protein and ash contents, while only muscle had an effect on the fat content. The mineral content of the bone, liver and *Longissimus thoracis et lumborum* muscle was also analyzed, and bone was found to be a rich source of calcium (highest concentration), whilst the liver had the highest concentration of iron. The chemical composition of the giraffe meat was such that it could be classified as lean meat.

## 1. Introduction

The global population is currently growing at such a rate that the population is expected to surpass nine billion within the next few decades [1]. Much of this growth is predicted to take place in Africa, a continent that is already struggling to feed its population, with southern Africa currently being a net importer of food, despite an economy that cannot support this [2]. Meat has a highly concentrated protein content that has a higher biological value than plant protein and an excellent amino acid profile [3] and contains other important nutrients and minerals, which also have a higher availability to humans [4]. Thus, meat is a very important part of the human diet. 

Since southern Africa is largely an arid region, conventional meat species are often not suited to the climate and cannot utilize the veld (natural vegetation) with its poor nutrient content; however, game species that are endemic to southern Africa are more suited to these conditions and well-adapted to the naturally available dietary plant species. Since there is a high diversity of game species from various taxonomic classifications, when investigating their potential as an alternative source of meat, it is necessary to investigate the nutritive value of the meat of each potential species in order to ensure that it fulfills the nutritional requirements of the consumers. Game meat has been found to have a low-fat content with a favorable mono- and polyunsaturated to saturated fatty acid ratio [3], which is desirable to the consumer, due to the relationship between fats in the food consumed and incidences of obesity or cardiovascular disease [5]. The nutritional value of meat is assessed primarily by determining the basic chemical composition in terms of moisture, protein, intramuscular fat (IMF) and ash content, which is an indication of the mineral content [6]. 

Meat contains many of the essential macro- and micro-minerals that are required for the human diet [7]. Many of these are found exclusively in animal tissue or in a more bioavailable form than in plant tissue, such as zinc (Zn) and magnesium (Mg) [8]. The iron (Fe) content of meat, especially red meat, also makes it an important part of the human diet, as the Fe found in meat is predominantly (50–60%) found in the haem form, which is more readily absorbed than the non-haem form found in plant tissue [9]. The mineral composition of giraffe meat has not yet been investigated. The liver is also known to have a relatively high mineral content; however, the mineral composition of giraffe liver has not yet been studied. Bone consists largely of minerals and is often ground into a meal which is used as a supplement in livestock feed, as it contains easily absorbable forms of the minerals required for bone growth in livestock. Bone consists of predominantly calcium and phosphorus lattice structures, with a wide spectrum of other minerals also involved in maintaining the rigidity of the bone. The density of giraffe bone in relation to that of African buffalo (*Syncerus caffer*) as another artiodactyl of similar mass has been investigated [10]. As the skeleton makes up a much greater proportion of the live weight in giraffe than in buffalo, the researchers investigated whether this affected the density of the bones in the giraffe in any way; however, the two species were found to have skeletons of similar density.

The potential of giraffe to provide meat in a sustainable manner that is also of an acceptable quality has been described previously [11,12]. Giraffe has been shown to have a favorable dress-out/dressing percentage (51.6–59.2% of dead weight) [11] with some of the main muscles having acceptable physical meat quality attributes [12]. This study aims to quantify the chemical composition of chilled (24 h post mortem) giraffe meat (eight different muscles) in terms of the moisture, protein, IMF and ash contents in order to develop a broader knowledge base on the composition and nutritional value of giraffe meat. It also aims to quantify the mineral composition of the bone, liver and *Longissimus thoracis et lumborum* (LTL) muscle.

## 2. Materials and Methods

### 2.1. Experimental Location and Animals

The harvesting of 15 young giraffes (8 male, 7 female; estimated average age of 3½ years old) on Mount Etjo farm in the Otjozondjupa region of Namibia as part of a cull that takes place every year, in order to curb the population growth as these giraffes have no natural predators on the farm, has been described [11]. The giraffes were culled by a head shot and then exsanguinated in the field (Ethical approval: ACU-2018-7366, Stellenbosch University; Shoot and sell permit number: 118690). They were then transported back to the abattoir where they were skinned, eviscerated and dressed as described [11]. The warm carcasses were quartered before being placed into the chiller (4 °C).

### 2.2. Processing and Sampling

Eight chilled muscles were removed from the left side of each carcass, namely the *Longissimus thoracis et lumborum* muscle (LTL), *Semimembranosus* muscle (SM), *Biceps femoris* muscle (BF), *Semitendinosus* muscle (ST), *Gluteus medius* muscle (GM), *Supraspinatus* muscle (SS), *Infraspinatus* muscle (IS) and *Psoas major* muscle (PM), for chemical analysis. The second last rib was removed and scraped clean of all soft tissue for mineral analyses whilst the liver was cut into three portions and the mid-portion taken for further analyses—these samples were all individually vacuum-packed and frozen at −20 °C until analyses. At deboning, approximately 24 h post mortem, a representative sample of approximately 100–200 g was cut from each muscle, vacuum-packed and frozen at −20 °C until analyses. Before analyses, all samples were removed from the freezer and placed into the fridge at ±4 °C to defrost for ±24 h. These samples were then removed from the vacuum bags, and the outer membranes and any other thick membranes were removed. The samples were cut into smaller pieces before being placed individually in a blender (Commercial Cutter FP 35, Omas Spa, Numana, Italy) and homogenized for approximately 1 min, ensuring that all moisture lost during thawing was added back into the bowl cutter for this. The samples were blended up until completely homogenous before chemical analyses; for the mineral determination, sub-samples were placed into small vacuum bags and refrozen until further analysis.

### 2.3. Chemical Analysis

The moisture and ash contents (g/100 g) of each muscle from each animal were determined as described in the AOAC Official Method 934.01 [13]. The lipid content of each sample was determined using the rapid solvent extraction method [14]. With a mixture of chloroform/methanol as the solvent, in a 1:2 (*v*/*v*) ratio, which is the recommended ratio for samples with a fat percentage lower than 5%, this was deemed the appropriate ratio, following a test run of all the muscle from one animal, using both the 1:2 and 2:1 ratios and finding no values higher than 5%. The protein content of each sample was determined from the filtrates that remained behind after the fat extraction, using a Leco Nitrogen/Protein Determinator (FP528—Leco Corporation, St. Joseph, MI, USA) with the method described in the AOAC Official Method 992.15 [15].

Homogenized liver and LTL samples, as well as defatted and incinerated bone samples from each giraffe, were used for mineral analysis. The bones were defatted using petroleum ether, before being incinerated for 24 h at 600 °C and crushed into a fine powder. All samples then underwent microwave digestion in Teflon vessels with Ultra Pure HNO_3_ and H_2_O_2_ using a MARS microwave digester with the settings as follows: power level: 1600 W, 100%; ramp time: 25 min; pressure: 800 psi; hold time: 10 min. The samples were cooled and diluted 10× in order to reduce the acid concentration. 

The samples underwent major, minor and trace element analysis, by inductively coupled plasma atomic emission spectroscopy (ICP-AES) and inductively coupled plasma mass spectrometry (ICP-MS). For ICP-AES, a Thermo iCAP 6000series (Thermo Scientific, Waltham, MA, USA) was used, with the following settings: RF power: 1350 W; carrier gas (argon): 0.65 L/min; aux gas (argon): 1.0 L/min; nebulizer: 2 ml/min micromist; internal standard used: 1 ppt Yttrium. For ICP-MS, an Agilent 7900 ICPMS (Santa Clara, CA, USA) was used with the following settings: RF power: 1600 W; carrier gas (argon): 0.83 L/min; sample depth: 10 mm; make-up gas: 0.15 L/min; helium flow: 5 mL/min; hydrogen flow: 6 mL/min; nebulizer: 0.4 mL/min micromist. 

The accuracy of all the chemical analyses in the laboratory was verified by a National interlaboratory scheme (AgriLASA: Agricultural Laboratory Association of South Africa) where blind samples are analyzed once every 3 months to control and ensure the accuracy and repeatability of the procedures used.

### 2.4. Statistical Analyses

The experimental design of this trial took the form of a split-plot design with sex as the main plot factor and muscle (LTL, SM, BF, ST, GM, SS, IS, PM) as the sub-plot factor. Statistica Version 13.4 (2018) R (lmer package) was used to perform a univariate analysis of variance (ANOVA) using the general linear model (GLM) procedures on the parameters for the proximate analyses (moisture, ash, IMF, protein) and mineral content. Deviations from normality were assessed by means of the Shapiro–Wilk test on the standardized residuals from the model [16]). Where observations diverged too far from the model value, they were removed as outliers. For the comparison of sex and muscle effects, Fisher’s least significant difference was calculated at the 5% significance level [16].

## 3. Results

There was a significant interaction between the effect of sex and muscle on the moisture (*p* = 0.044), protein (*p* = 0.045) and ash contents (*p* = 0.042) of giraffe meat, however not on the IMF content (*p* = 0.790) as seen in Table 1. Due to these interactions, the proximate composition parameter values of each muscle are reported separately for both sexes in Table 2. The moisture content was significantly higher in males than in females for the ST (male: 78.0 ± 0.22 g/100 g; female: 77.0 ± 0.38 g/100 g) and the GM (male: 77.9 ± 0.18 g/100 g; female: 76.5 ± 0.37 g/100 g), while the protein content was higher in females than males for the ST (male: 20.6 ± 0.23 g/100 g; female: 21.4 ± 0.44 g/100 g) and GM (male: 20.2 ± 0.23 g/100 g; female: 21.3 ± 0.28 g/100 g). The IMF content was higher in females than in males only for the SS muscle and was the lowest in the ST (1.1 ± 0.08 g/100 g), while the LTL, GM, SS and PM had the highest IMF contents. The ash content was significantly higher in females than in males for the GM (male: 1.1 ± 0.01 g/100 g; female: 1.2 ± 0.04 g/100 g) and the SS (male: 1.0 ± 0.01 g/100 g; female: 1.1 ± 0.02 g/100 g).

The mineral composition was analyzed separately for the bone, liver and meat (LTL muscle only). Some of the minerals were not detected in all body parts (Table 3). While arsenic (As) was not present in levels above the lowest detection limits (LODs) in any body part, tin (Sn) and silicon (Si) were not found in levels above the LODs for the liver or the LTL, and silver (Ag), cadmium (Cd), mercury (Hg) and lead (Pb), while found in the bone and liver, were not found in levels above the LODs in the LTL. Sex had an effect only on the lead (Pb) levels in the bone, with higher levels in males than the females. For the liver, sex influenced the silver (Ag) levels, with a higher content in the females than the males. The LTL had differences between the sexes for several minerals; barium (Ba), aluminum (Al), vanadium (V), copper (Cu), zinc (Zn) and sodium (Na) all had a higher concentration in males than in females. 

## 4. Discussion

The objective of this study was to determine the influence of sex and muscle type on the chemical meat quality of giraffe, through proximate analysis. A secondary objective was to compare the mineral composition of the LTL muscle, rib bone and liver. In general, lean skeletal muscle is made up of approximately 75% moisture, 20% protein, 1–10% IMF and 1% carbohydrates, vitamins and minerals, which are usually quantified as the ash content [3]. These percentage compositions vary by species, sex and muscle, as well as the diet that the animals consume. For giraffe, the average composition across sex and muscle was 77.2 ± 0.09% moisture, 20.8 ± 0.09% protein, 1.4 ± 0.03% IMF and 1.1 ± 0.01% ash (Table 1 and Table 2), which is in alignment with that of other lean game meat, with slightly higher moisture content and IMF on the lower end of the range. Giraffe meat was very lean with no visible intramuscular fat, as noted during the data collection process. Poor water-holding capacity was reported [12]; however, the meat in this study has a slightly higher moisture content than lean meat of other species [17].

The moisture content of the giraffe meat ranged from 76.4 to 78.3 g/100 g, with the LTL, SM and BF having the lowest moisture contents, while the SS and IS had the highest moisture contents. There is little research on the various muscles of game meat; however, many studies report the chemical composition of only the LTL. The moisture content of the LTL of the giraffe (76.5 ± 0.19 g/100 g) is similar to the moisture content of the LTL of the eland (75.6–77.8 g/100 g [18]) and the blue wildebeest (75.9–78.5 g/100 g [19]). The LTL of the giraffe, however, had a higher moisture content than that of impala (75.5 ± 0.12 g/100 g [20]), springbok (65.3–65.8 g/100 g [21,22]), kudu (75.7–75.8 g/100 g [23]) and blesbok (73.9–76.1 g/100 g [24]). 

The protein content of the giraffe meat ranged from 19.9 to 21.5 g/100 g, with a significant interaction between the sex and muscle. The SS and the IS had the lowest protein across the sexes, while the LTL, SM, ST, BF and PM had the highest protein content across the sexes. The ST and GM both had significantly higher protein contents in females than in males which correlates with a lower moisture content in these two muscles in the females than in the males, and they were the only two muscles to differ for sex for these parameters. If one compares the protein content of the LTL (21.4 ± 0.25 g/100 g) with that of other game species, it compares favorably with protein content from most other game species with impala (22.5 ± 0.15 g/100 g [20]), ostrich (22.2 ± 1.13 g/100g [25]), blesbok (19.0–23.1 g/100 g [24,26]) and blue wildebeest (19.3–22.3 g/100 g [19]), however substantially lower than the values recorded for springbok (31.1 ± 0.45 g/100 g [21]). Game meat typically has a favorable protein content relative to traditional livestock species, although this may be due to a higher IMF percentage in domestic species, which makes it a healthy alternative to commercially produced red meat. 

There was no significant interaction between the effect of the sex and the muscle on the IMF content of the giraffe meat, which ranged from 1.0 to 1.7 g/100 g. Sex did not have an effect on the fat content, despite many studies finding females to have a higher fat content than males (fallow deer [27], impala [20,23], eland [28], springbok [21], blesbok [26], black wildebeest [29] and blue wildebeest [19]). This may be because the giraffes were pubescent and not exhibiting full sexual dimorphisms yet [11]. Muscle did have an effect on the fat content with the LTL, GM, SS and PM having the highest fat contents and the ST the lowest. When comparing the IMF of the LTL (1.6 ± 0.09 g/100 g) to that of other game species, it is similar to that of impala (1.7 ± 0.06 g/100 g [20]), ostrich (1.6 ± 0.60 g/100 g [25]), eland (1.45–1.48 g/100 g [18]), kudu (1.48–1.497 g/100 g [23]) and blue wildebeest (1.6–2.1 g/100 g [19]) but lower than that of blesbok (2.3–3.4 g/100 g [24]). IMF is very season- and location-dependent; these factors should be considered when comparing species, and this is especially important between sexes as males will have a lower fat content than usual during rut, while females’ fat content will fluctuate during gestation. The fat content of game species is generally considerably lower than that of the conventional meat species. 

The ash content is an indication of the mineral or the inorganic components of the meat. There was an interaction between the effects of sex and muscle for the ash content. The ash content of the giraffe meat ranged from 1.0 to 1.2 g/100 g, and therefore, despite the differences, the magnitude was very small. While the GM and SS had significantly higher ash contents in females than in males, the other muscles did not differ for sex. The SM, ST and PM had the highest ash content across the sexes and the SS and IS the lowest. When comparing the ash content of the LTL (1.1 ± 0.02 g/100 g) to other game species, it was similar to impala (1.24 ± 0.01 g/100 g [20]), blue wildebeest (0.99–1.1 g/100 g [19]), eland (1.0–1.1 g/100 g [18]) and kudu (1.1–1.2 g/100 g [23]). 

The mineral composition of the bone, liver and meat was compared between sexes (Table 3), and there were differences for specific minerals between the sexes for each body part. The mineral content of bone is determined by a balance between bone formation and resorption, which is affected by age, diet and physiological state. The diet of giraffe contains a ratio of calcium to phosphorus (Ca:P) of approximately 7.7:1 [30,31], which is much lower than it would be for grazers, where it is normally closer to 2:1. With phosphorus levels this low in the diet, it would result in clinical signs of phosphorus deficiency, such as pica, in cattle [30,31], a form of which, osteophagia, has been broadly documented in giraffe. Giraffes have been observed chewing both other giraffe bones as well as those of other species [32,33,34,35], which suggests they are deficient in phosphorus. In our study, the bone of the giraffe had high levels of calcium, strontium, phosphorus, barium and zinc (Table 3). Calcium and phosphorus generally occur in a 2:1 ratio which was also found to be the case for the giraffe in this study and therefore indicates no P deficiency. The only significant difference between the two sexes in the bone minerals was for the levels of lead (*p* = 0.037) which was twice as high in males (204.2 ± 34.23 µg/kg) as in the females (109.9 ± 18.47 µg/kg), which may be due to diet, metabolism or physiological differences between the sexes [7]. The bone had higher levels of most of the macro-minerals than the liver and meat, having higher levels of zinc, magnesium, sodium and phosphorus, while the liver had the highest levels of iron, and the meat had the highest potassium levels.

The liver had a significantly higher ash content than the other organs (2.0 ± 0.14 g/100 g) which is also significantly higher than the ash content of the meat of the giraffe (1.00–1.21 g/100 g; Table 3). As ash is a measure of the mineral content, this means that the liver has a higher mineral content than the meat. The liver had very high iron, zinc and copper levels. The only significant difference between sexes was for the levels of silver (*p* = 0.042), which was higher in females (22.3 ± 7.50 µg/kg) than in males (6.4 ± 0.89 µg/kg), which may be due to differences in diet and metabolism of physiological functions between the two sexes.

Meat contains many essential macro-minerals, specifically high levels of potassium and phosphorus as well as moderate sodium and magnesium levels, with a lower content of calcium [4]. Meat also contains a range of essential micro-minerals including iron, copper, zinc, cobalt, manganese, selenium and molybdenum, of which some are only found in muscle tissue or in a more bioavailable form than in plant tissues [4]. There are limited studies on the mineral composition of game species, and those that have been conducted are generally limited to only a few minerals. No differences between sexes for any of the minerals analyzed for springbok were reported [22]; however, the levels of boron, aluminum, vanadium, copper, zinc and sodium were found to be higher in males than in females for the giraffe. Higher contents of calcium, potassium, magnesium, sodium and phosphorus than in the LTL of the giraffe were found in the same muscle in springbok [22]. In contrast, lower levels of iron, copper and zinc were reported for the springbok [22] than found in giraffe. Iron, copper and zinc are all important to the human diet, making giraffe meat a good source of these micro-minerals as well as other macro- and micro-minerals.

## 5. Conclusions

This study aimed to determine the effect of sex and muscle on the proximate composition of giraffe meat, as well as the effect of sex on the mineral composition of the bone, liver and meat of the giraffe. Significant interactions were found between sex and muscle for all proximate parameters (moisture, protein and ash), other than the IMF content, for which only muscle had an effect. Nonetheless, the protein content of the giraffe meat ranged from 19.9 to 21.5 g/100 g between the different muscles whilst the IMF ranged from 1.0 to 1.7 g/100 g, making giraffe meat a dense protein of the lean-meat type. As age is known to influence the chemical composition of different muscles in most animals, it is recommended to repeat the study with giraffes of different age groups to quantify the effect that age has on the proximate composition. There was a sex effect on a few of the minerals of the bone, liver and LTL of the giraffe, which was most likely due to dietary, metabolic or physiological differences between the sexes. The bone had a calcium to phosphorus ratio of 2:1 which is similar to the bone of other species. The liver of the giraffe was found to be high in manganese, and the liver and meat were both found to contain high levels of iron which are essential to the human diet. Further study is recommended on the effect of age on the mineral composition of the giraffe bone, liver and meat. Giraffe meat has a high moisture content, a low IMF and a high protein content on a par with those of other game species, containing many macro- and micro-minerals that are required in the human diet, making it a healthy meat to consume. 

## Figures and Tables

**Table 1:** Level of statistical significance (*p*-values) for the main effects of sex and muscle and their interaction for the proximate composition (g/100 g) of eight muscles from giraffe (n = 15).

Parameter	*p*-Value		
	Sex	Muscle	Sex × Muscle
Moisture (%)	0.046	<0.001	0.044
Protein (%)	0.226	<0.001	0.045
Intramuscular fat content (%)	0.100	<0.001	0.790
Ash (%)	0.050	<0.001	0.042

**Table 2:** Means (±standard error) of the proximate composition (g/100 g) of eight muscles from giraffe (n = 15) as influenced by sex and muscle. Both main effects and interactions were included for all parameters.

Parameter (g/100 g)	Muscle	Pooled for Sex ^#^	Sex *	
		(n = 15)	Male (n = 7)	Female (n = 8)
Moisture	LTL	76.5 ^e^ ± 0.19	76.6 ^c^ ± 0.29	76.4 ^c^ ± 0.26
	SM	76.8 ^de^ ± 0.19	77.0 ^bc^ ± 0.24	76.6 ^c^ ± 0.30
	BF	77.0 ^de^ ± 0.24	77.0 ^bc^ ± 0.41	76.9 ^c^ ± 0.26
	ST	77.5 ^bc^ ± 0.24	78.0 ^a^ ± 0.22	77.0 ^c^ ± 0.38
	GM	77.2 ^cd^ ± 0.26	77.9 ^a^ ± 0.18	76.5 ^c^ ± 0.37
	SS	77.9 ^ab^ ± 0.17	78.1 ^a^ ± 0.26	77.7 ^ab^ ± 0.21
	IS	78.1 ^a^ ± 0.16	78.3 ^a^ ± 0.20	77.8 ^a^ ± 0.23
	PM	76.9 ^de^ ± 0.20	76.9 ^c^ ± 0.36	76.9 ^c^ ± 0.16
Pooled for muscle		77.2 ± 0.09	77.5 ^a^ ± 0.12	77.0 ^b^ ± 0.11
Protein	LTL	21.4 ^a^ ± 0.25	21.4 ^a^ ± 0.41	21.3 ^ab^ ± 0.29
	SM	21.4 ^a^ ± 0.16	21.2 ^a^ ± 0.27	21.5 ^a^ ± 0.15
	BF	21.1 ^ab^ ± 0.24	21.1 ^ab^ ± 0.41	21.2 ^ab^ ± 0.28
	ST	21.0 ^ab^ ± 0.26	20.6 ^bc^ ± 0.23	21.4 ^a^ ± 0.44
	GM	20.7 ^b^ ± 0.23	20.2 ^cd^ ± 0.23	21.3 ^ab^ ± 0.28
	SS	19.9 ^c^ ± 0.20	19.9 ^d^ ± 0.30	20.0 ^cd^ ± 0.26
	IS	20.0 ^c^ ± 0.16	19.9 ^d^ ± 0.22	20.2 ^cd^ ± 0.22
	PM	21.1 ^ab^ ± 0.24	21.2 ^a^ ± 0.41	21.0 ^ab^ ± 0.27
Pooled for muscle		20.8 ± 0.09	20.7 ± 0.13	21.0 ± 0.12
IMF	LTL	1.6 ^a^ ± 0.09	1.4 ^abcde^ ± 0.13	1.7 ^a^ ± 0.11
	SM	1.3 ^c^ ± 0.09	1.2 ^de^ ± 0.08	1.5 ^cd^ ± 0.16
	BF	1.3 ^c^ ± 0.08	1.3 ^de^ ± 0.08	1.4 ^d^ ± 0.14
	ST	1.1 ^d^ ± 0.08	1.0 ^f^ ± 0.08	1.2 ^ef^ ± 0.13
	GM	1.5 ^ab^ ± 0.10	1.4 ^bcde^ ± 0.12	1.7 ^abc^ ± 0.16
	SS	1.5 ^abc^ ± 0.11	1.3 ^de^ ± 0.11	1.7 ^ab^ ± 0.17
	IS	1.4 ^bc^ ± 0.07	1.3 ^de^ ± 0.10	1.5 ^abcd^ ± 0.09
	PM	1.4 ^abc^ ± 0.10	1.3 ^bcde^ ± 0.09	1.5 ^abcd^ ± 0.19
Pooled for muscle		1.4 ± 0.03	1.3 ± 0.04	1.5 ± 0.05
Ash	LTL	1.1 ^b^ ± 0.02	1.1 ^cde^ ± 0.01	1.1 ^bcde^ ± 0.04
	SM	1.2 ^ab^ ± 0.02	1.2 ^ab^ ± 0.03	1.1 ^bcde^ ± 0.02
	BF	1.1 ^b^ ± 0.02	1.1 ^def^ ± 0.01	1.2 ^abcd^ ± 0.03
	ST	1.2 ^ab^ ± 0.01	1.1 ^cde^ ± 0.01	1.2 ^abc^ ± 0.02
	GM	1.1 ^b^ ± 0.02	1.1 ^ef^ ± 0.01	1.2 ^abcd^ ± 0.04
	SS	1.0 ^c^ ± 0.02	1.0 ^g^ ± 0.01	1.1 ^ef^ ± 0.02
	IS	1.0 ^c^ ± 0.02	1.0 ^g^ ± 0.03	1.0 ^fg^ ± 0.02
	PM	1.2 ^a^ ± 0.02	1.2 ^abc^ ± 0.02	1.2 ^a^ ± 0.04
Pooled for muscle		1.1 ± 0.01	1.1 ± 0.01	1.1 ± 0.01

LTL = Longissimus thoracis et lumborum, SM = semimembranosus, BF = biceps femoris, ST = semitendinosus, GM = gluteus medius, SS = supraspinatus, IS = infraspinatus, PM = Psoas major. ^# a–e^ Means with different superscripts within a parameter pooled for sex differ between muscles (*p* ≤ 0.05). * ^a–g^ Means with different superscripts within a parameter for muscle differ between sexes (*p* ≤ 0.05).

**Table 3:** The major, minor and trace element content of the bone, liver and LTL of giraffe, as influenced by sex.

Mineral	…/kg Tissue	Limit of Detection (…/kg Tissue)	Bone			Liver			LTL		
			Male (n = 8)	Female (n = 7)	*p*-Value	Male (n = 8)	Female (n = 7)	*p*-Value	Male (n = 8)	Female (n = 7)	*p*-Value
Ba	µg	139.6	9812.6 ± 584.73	8844.7 ± 561.87	0.257	1023.8 ± 86.95	941.5 ± 56.96	0.457	1023.0 ± 87.71	717.1 ± 64.16	0.017
Al	µg	156.2	11,086.0 ± 5492.62	3644.7 ± 596.64	0.231	997.4 ± 209.21	920.2 ± 174.78	0.785	1058.5 ± 134.17	482.1 ± 64.07	0.003
V	µg	1.1	18.1 ± 6.35	7.7 ± 0.45	0.151	11.4 ± 5.58	5.2 ± 0.26	0.322	4.4 ± 0.40	3.2 ± 0.21	0.017
Cr	µg	28.0	887.1 ± 491.67	196.4 ± 23.11	0.214	391.4 ± 99.76	248.0 ± 70.75	0.275	272.3 ± 139.34	137.2 ± 72.87 *^(5)^	0.487
Mn	µg	13.2	578.8 ± 66.30	504.8 ± 38.27	0.370	2384.1 ± 80.44	2277.1 ± 87.83	0.384	123.7 ± 15.86	85.4 ± 5.66	0.051
Fe	µg	56.5	23,662.1 ± 5133.19	16,764.2 ± 2631.28	0.273	73,083.3 ± 4853.47	87,123.3 ± 13,637.47	0.325	15,976.8 ± 1144.22	13,786.9 ± 1188.21	0.208
Co	µg	1.1	13.0 ± 2.63	9.6 ± 0.72	0.263	86.1 ± 3.17	85.7 ± 2.41	0.920	3.7 ± 0.96	3.5 ± 1.12	0.866
Ni	µg	0.7	202.2 ± 54.12	231.1 ± 40.04	0.682	2.4 ± 55.79	118.6 ± 35.68	0.241	131.6 ± 67.63	581.7 ± 385.71	0.241
Cu	µg	5.3	1094.2 ± 190.72	769.7 ± 133.56	0.199	21,874.3 ± 2479.27	19,176.5 ± 2363.69	0.449	1063.3 ± 74.73	805.5 ± 24.20	0.009
Zn	µg	5.5	130,020.6 ± 3828.56	12,3261.9 ± 4021.0	0.246	35,535.8 ± 848.03	36,057.7 ± 1255.54	0.730	30,107.7 ± 2064.10	23,394.3 ± 1729.44	0.029
As	µg	5.1	-	-		-	-		-	-	
Se	µg	1.6	25.9 ± 3.24	23.7 ± 1.40	0.563	313.4 ± 15.72	334.8 ± 18.75	0.394	98.0 ± 3.81	116.4 ± 18.45	0.316
Sr	µg	14.7	230,856.2 ± 18,607.24	230,973.1 ± 24,689.38	0.997	31.4 ± 3.15	61.6 ± 22.48	0.178	28.2 ± 4.04	21.5 ± 1.70	0.173
Mo	µg	0.7	125.7 ± 13.65	98.6 ± 18.79	0.256	993.2 ± 32.78	907.26 ± 37.67	0.107	21.3 ± 4.61	10.77 ± 1.07	0.057
Ag	µg	4.0	63.7 ± 14.50	28.2 ± 8.55	0.064	6.4 ± 0.89	22.3 ± 7.50	0.042	-	-	
Cd	µg	1.6	8.0 ± 3.45 *^(6)^	2.1 ± 0.14 *^(5)^	0.158	9.3 ± 1.78	6.0 ± 0.89	0.135	-	-	
Sn	µg	2.4	11.2 ± 3.02	11.7 ± 5.34	0.934	-	-		-	-	
Sb	µg	0.7	33.2 ± 13.85	15.1 ± 2.80	0.253	10.1 ± 4.63	4.5 ± 2.46	0.328	4.1 ± 0.75	2.9 ± 0.79	0.274
Ba	µg	0.9	185,834.1 ± 8203.37	189,793.2 ± 11,277.81	0.777	32.7 ± 2.77	74.1 ± 36.20	0.242	22.3 ± 3.37	14.4 ± 1.27	0.060
Hg	µg	0.7	2.6 ± 0.55	1.5 ± 0.27 *^(6)^	0.125	5.8 ± 0.72	6.0 ± 0.79	0.858	-	-	
Pb	µg	5.1	204.2 ± 34.23	109.9 ± 18.47	0.037	12.9 ± 1.85	8.9 ± 0.89	0.082	-	-	
Ca	mg	10	436,252.1 ± 2254.85	431,341.0 ± 3409.02	0.240	45.1 ± 1.95	94.2 ± 39.62	0.206	41.3 ± 4.10	35.6 ± 0.74	0.228
K	mg	10	1374.0 ± 127.92	1314.8 ± 143.42	0.762	3255.8 ± 48.72	3294.01 ± 112.49	0.749	4100.81 ± 43.82	4007.3 ± 70.37	0.267
Mg	mg	10	9363.5 ± 130.36	9135.1 ± 133.52	0.244	162.3 ± 2.08	164.28 ± 5.07	0.716	250.0 ± 2.35	243.9 ± 3.67	0.171
Na	mg	10	11,670.7 ± 244.51	11,918.1 ± 91.98	0.387	723.8 ± 24.29	780.1 ± 46.10	0.282	381.5 ± 11.11	343.5 ± 6.18	0.013
P	mg	10	212,082.6 ± 634.28	209,584.6 ± 1074.67	0.059	3515.5 ± 57.90	3555.6 ± 91.58	0.710	2261.6 ± 22.68	2230.5 ± 26.70	0.388
Si	mg	5	34.4 ± 13.31	16.5 ± 0.68	0.233	-	-		-	-	

Values with * have values below the LOD; ^(#)^ number of values above the limit of detection; - indicates too many values were below LOD for statistical significance.

## Data Availability

The data presented in this study are available on request from the corresponding author. The data are not publicly available due to privacy restrictions.
